# Outcomes of remotely delivered behavioral insomnia interventions for children and adolescents: systematic review of randomized controlled trials

**DOI:** 10.3389/frsle.2023.1261142

**Published:** 2024-01-11

**Authors:** Suncica Lah, Thanh Vinh Cao

**Affiliations:** School of Psychology, The University of Sydney, Sydney, NSW, Australia

**Keywords:** insomnia, sleep, treatment, remote delivery, children, adolescents

## Abstract

Pediatric insomnia is common and can be effectively treated with behavioral therapies delivered face-to face. Such treatments could also improve children's mood, cognition, and quality of life, and caregivers' wellbeing. There is a discrepancy between high needs and limited access to pediatric behavioral insomnia treatments, which could be improved by provision of technology enhanced interventions. No study reviewed outcomes of randomized controlled trials (RCTs) of remotely delivered psychological treatments for pediatric insomnia. The current study aimed to examine (i) the outcomes of remotely delivered RCTs for pediatric insomnia/insomnia symptoms and (ii) whether gains made in treatment extend to functional correlates. We conducted a systematic review according to Cochrane and PRISMA guidelines. PsychINFO, PubMed/Medline and Cochrane CENTRAL databases were searched for RCTs reporting on remotely delivered behavioral treatments for insomnia and insomnia symptoms. Data was abstracted and the risk of bias were assessed in November 2022 and November 2023. Seven RCTs (nine manuscripts) involving 786 participants, with the mean age from 19.3 months to 16.9 years, were identified. Four different treatments were used. Risk of bias ranged from low to high and was the highest for the randomization process. Across studies, significant improvements were found in some (but not all) sleep parameters, namely: sleep quality and sleep efficacy on questionnaires and on actigraphy, despite heterogeneity of the treatments used, age of participants and instruments employed to assess outcomes. Improvements gained in treatments delivered remotely was compared to treatments delivered face-to-face in 3 studies and were found to be comparable or slightly lower. No worsening was observed on either objective or subjective measures of sleep, except for sleep onset latency and wake after sleep onset that improved on questionnaires but worsened on actigraphy in one study each. Children's mood improved across studies on parent and self-report measures. Other possible functional gains were understudied. Our study provides preliminary evidence of improved sleep following remotely delivered behavioral treatments for pediatric insomnia, and improvements in children's mood. Further research is needed to develop individualized treatments that will cater for different developmental needs and types of insomnia symptoms and examine not only group but also individual outcomes.

## 1 Introduction

Insomnia is characterized by poor sleep quality or quantity in relation to one or more of the following symptoms: persistent difficulties initiating sleep, maintaining sleep or early morning awakening (American Psychiatric Association, 2013). These sleep difficulties are present despite good opportunities for sleep and impair some aspects of daytime functioning (American Academy of Sleep Medicine, 2014). In children, a term “behavioral insomnia” refers to difficulties falling asleep or staying asleep without caregivers' intervention (Morgenthaler et al., 2006; Owens and Mindell, 2011). The sleep difficulties are perceived as a problem by either the child or a parent and impact daily functioning of the child or family (Owens, 2007). In adolescents, the adult definition can be used (Owens, 2007). Insomnia and insomnia symptoms have a prevalence rate of up to 25–40% in children (Medalie et al., 2021) and up to 40% in adolescents (Chung et al., 2014). In children and adolescents, disturbed sleep is associated with high rates of anxiety and depression (Sivertsen et al., 2021) and social-emotional problems (Hysing et al., 2016), greater behavioral problems (Gregory and O'Connor, 2002; Van Veen et al., 2021), lower academic performance (Fallone et al., 2005; Zhang et al., 2022), reduced cognitive functioning (Bruni et al., 2020; Li et al., 2022) and lower quality of life (Combs et al., 2016; Magee et al., 2017). Moreover, disturbed sleep can impact caregivers' wellbeing: sleep, daytime functioning, and health (Martin et al., 2007; Meltzer and Mindell, 2007). Given the high prevalence and significant functional correlates of insomnia, it is imperative to provide effective treatments.

Behavioral and psychological treatments are the recommended first line of treatment for insomnia by the Australian Sleep Association (Ree et al., 2017) and the American Academy of Sleep Medicine (Morgenthaler et al., 2006; Qaseem et al., 2016). Behavioral and psychological treatments provide reliable, clinically significant improvements in sleep, and are more effective in improving sleep in the long-term than pharmacological treatments (Jacobs et al., 2004). Caregivers perceive children having sleep problems when children wake up overnight, have difficulties sleeping alone, and have difficulties falling asleep (Williamson et al., 2019). While these three sleep behaviors are reported across ages, their frequency, and the magnitude of their relationship with parental reported sleep problems varies with age. In young children and toddlers, night waking and bedtime refusal, involve support to parents to establish bedtime routines, set limits, provide positive reinforcement, and implement gradual extinction strategies (Tikotzky and Sadeh, 2010; Owens and Mindell, 2011). Older children and adolescents are more likely to experience difficulties falling asleep and maintaining sleep due to worries, heightened physiological arousal and disturbed circadian rhythm (Donskoy and Loghmanee, 2018). Treatment that typically involves cognitive behavioral therapy for insomnia (CBT-I) is delivered to children/adolescents, with parents often playing a supporting role (Vriend and Corkum, 2011). A cognitive component of CBT-I refers to reframing of unhelpful thoughts about sleep, which changes how patients feel about sleep and enables behavioral changes around sleep (Paine and Gradisar, 2011). Stimulus control and sleep restrictions are the crucial behavioral components of CBT-I, which typically also includes psychoeducation about sleep and sleep hygiene (Perlis et al., 2004; Lewin and Huntley, 2017). Recent studies have found that CBT-I is effective in treating insomnia in children and adolescents (Tikotzky and Sadeh, 2010; Aslund et al., 2018). Specifically, CBT-I was found to improve sleep onset latency (SOL), sleep efficiency (SE) (Ma et al., 2018), total sleep time (TST) and wake after sleep onset (WASO) (Blake et al., 2017). Moreover, behavioral interventions also improve sleep of children and adolescents with neurological and neurodevelopmental disorders (Phillips et al., 2020; Lah et al., 2021), lessen behavioral difficulties, inattention, and hyperactivity, and fatigue. Traditionally, behavioral sleep interventions and CBT-I have been delivered in individual face-to-face sessions involving a trained therapist and a patient. While effective, access to face-to-face therapies is limited by the time required to travel to and from treatment sessions, costs of attending individual therapy sessions, and availability of trained therapists. The COVID-19 pandemic has hindered the delivery of face-to-face interventions generally and escalated a discrepancy between high needs for insomnia treatment and limited access to psychological treatments for insomnia.

Accessibility of treatment is improved, and cost is reduced when CBT-I is delivered via technology enhanced interventions, through websites, mobile applications, telephone consultations or telehealth consultations (Mack and Rybarczyk, 2011; Thiart et al., 2016). These remotely delivered treatments, however, require supporting technology, which may not be available to all people (especially to people with limited resources, and people with different cultural backgrounds), although basic telephone service is typically widely available (McCurry et al., 2020). Treatments delivered via websites and mobile applications are potentially most scalable and may be particularly appealing and beneficial for “digital natives” (Prensky, 2001a,b): those born after 1980. In adults, RCTs of fully automated digital CBT-I were found to be effective, with effect sizes comparable to automated programs delivered with therapist support (Luik et al., 2017). However, there is limited research into the use of the remotely delivered treatment of insomnia for children and adolescents. To date, to our knowledge, one systematic review examined the efficacy and effectiveness of a digitally delivered (web-based or mobile phone application) CBT-I for youth (Werner-Seidler et al., 2023). The digitally delivered CBT-I improved SE, SOL, TST and subjective sleep quality. While informative, this review included 3 manuscripts of which only one involved an RCT and the review was limited to youth aged 12 to 24 years. The findings of the review could not be extended to younger children due to the disparity in psychological and cognitive development of youth and children that mandate different approaches to treatments of insomnia. Furthermore, the review was limited to digitally delivered interventions, which may differ in their effectiveness from telehealth interventions that are delivered remotely by a therapist. Given the recent advances in technology, and the COVID-19 pandemic, developing an evidence base for remotely delivered interventions is important. Such evidence base should consider not only studies involving typically developing children, but also studies involving clinical pediatric populations, as elimination of travel time and increased accessibility to treatment, and improved sleep, may ease the already high burden of care in clinical populations. Moreover, behavioral interventions for sleep may also result in secondary gains, such as reduction of behavioral difficulties in children with neurodevelopmental and neurological disorders (Phillips et al., 2020), which can further decrease the burden of care and increase carers' quality of life. The primary aim of the current study is to examine and synthesize findings in relation to sleep outcomes of published RCTs that include remotely delivered psychological treatments for child or adolescent insomnia symptoms or behavioral sleep disturbances. The secondary aim is to examine findings related to functional correlates, such as mood and behavior, cognition and academic achievement and quality of life in children and adolescents, and wellbeing of their caregivers.

## 2 Methods

This systematic review was guided by established guidelines from the preferred reporting items for systematic review and meta-analysis (PRISMA) protocols (Moher et al., 2015; Supplementary Table 1, Prisma 2020 Checklist, Page et al., 2021) and Cochrane Handbook (Higgins et al., 2022).

### 2.1 Study selection

#### 2.1.1 Inclusion criteria

(1) Peer-reviewed publication in the English language.
(2) Human participants aged 0 to 18 years.
(3) Diagnosed with insomnia or presented with insomnia symptoms (difficulties initiating sleep, maintaining sleep or early morning awakening) or behavioral sleep disturbances (difficulties falling asleep or staying asleep without caregivers' intervention) alone or concurrently with co-morbid disorders. No restriction was imposed on the criteria and methods used to identify insomnia or insomnia symptoms.
(4) RCTs involving behavioral interventions/CBT-I targeting insomnia, insomnia symptoms or behavioral sleep disturbances, and delivered remotely.
(5) Reported original data on sleep outcomes at baseline and post-intervention.

#### 2.1.2 Exclusion criteria

(1) Review papers or meta-analyses.
(2) Mixed age ranges with data not reported separately for children or a mean age-range of participants > 18 years.
(3) Pre- and post-intervention sleep data not reported.

### 2.2 Search strategy

A search of the databases PsychINFO and PubMed/MEDLINE via OVID, and Cochrane Central Register of Controlled Trials (CENTRAL), was conducted on 18 October 2022 and updated on 28 October and 1 November 2023. Database search terms and exploded medical subject headings, where relevant, included “sleep” AND “intervention” AND “online,” along with variations and permutations of these term. The full search strategy can be found in the Supplementary Table 2.

The searches conducted on PsychINFO and PubMed/MEDLINE via OVID were restricted to papers in the English Language, and human participants aged 0–18 years old. No date limits were set. The reference list of reviews identified in the search and empirical studies that met inclusion criteria were manually searched to identify further relevant articles that were not included in the databases, as per the ancestry method.

### 2.3 Selection of studies

All studies retrieved in the searches were tracked through a reference management software (EndNote). The titles and abstracts of retrieved references were screened by one reviewer (TVC). Studies that met the inclusion/exclusion criteria and studies that did not provide sufficient information to decide whether they meet criteria, were subsequently reviewed at the full-text stage. Full text manuscripts were reviewed by two authors independently, and any discrepancies were resolved via consensus. Data extraction criteria and data extraction form were developed by one author (SL) and piloted by both authors. Data was abstracted by one author (TVC) and verified by another (SL) in November 2022 and November 2023.

### 2.4 Quality ratings of selected papers

The risk of bias was independently assessed by two authors using the Revised Cochrane Risk of Bias Tool for RCTs (ROB 2, Sterne et al., 2019). Any disagreements between reviewers were resolved by consensus. In circumstances where insufficient detail was provided to make a clear risk of bias judgement, authors of included papers were contacted to obtain further information.

The ROB 2 assesses a risk of bias in 5 domains: randomization process, deviations from intended interventions, missing outcome data, measurement of the outcome and selection of the reported result. Each domain is evaluated via a number of signaling questions. On completion, the ROB 2 algorithm provides a proposed risk of bias for each domain as well as the overall risk of bias for each study. The risk of bias is categorized as: low, high, or unclear.

## 3 Results

**Figure 1:**
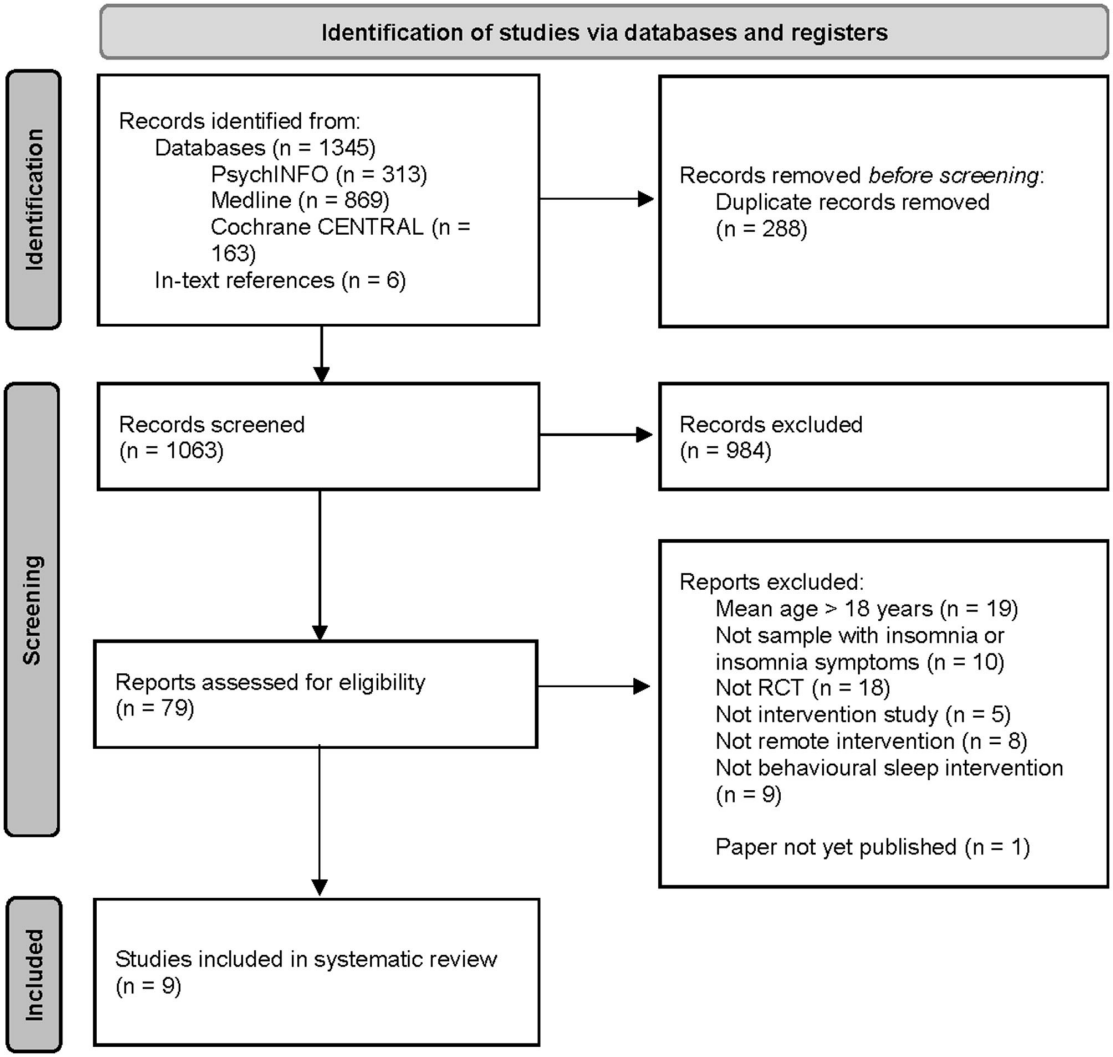
PRISMA Flowchart of studies selection process.

Details of study selection are fully provided in Figure 1. The electronic searches yielded 1,354 records. In addition, six records were identified through the ancestry method. On removal of duplicates, titles, and abstracts of 1,063 records that remained were screened against the inclusion/exclusion criteria. On completion of screening, 79 records that either met the inclusion criteria or did not provide sufficient information in the title and abstract to determine whether they meet the inclusion criteria underwent a full-text review. A total of 70 articles were subsequently excluded for the following reasons: (i) mean age of participants > 18 years old (*n* = 19), (ii) participants did not have either insomnia or insomnia symptoms (*n* = 10), (iii) the intervention did not primarily target insomnia, insomnia symptoms or behavioral sleep disturbances (*n* = 9), (iv) the study did not include an intervention component (*n* = 5), (v) the intervention was not delivered remotely (*n* = 8), (vi) the full-text manuscript was not yet published (*n* = 1), and (vi) the study was not an RCT (*n* = 18). On completion of the selection, 9 manuscripts (reporting on 7 studies) met the inclusion criteria for the systematic review.

### 3.1 Study and patient characteristics

Please see Table 1 for details of included studies. Of the 7 RCTs, 3 were conducted by the same research team from Netherlands. The remaining 4 RCTs were conducted by different research teams, 2 teams from the United States of America, 1 team from Canada, and 1 from Australia.

**Table 1:** Study characteristics.

**Study and design**		**Participants**					
**References (Country)**	**RCT design (control) and FU**	**Total** ***N***	*M*_age_ **(range)**	**Sex**, ***n***	**Race/ethnicity**, ***n***	**Sleep problems**	**Comorbidities**
Mindell et al. (2011a,b)^#^ (USA)	3-arm parallel group (TAU) 1-year FU	264	19.3 months (6–36 months)	131 M 133 F	NR	Mothers of children with parent-identified “small” to “serious” sleep problem and bedtime difficulties	N/A
de Bruin et al. (2014) (Netherlands)	2-arm parallel group (Group Therapy) 2-month FU	26	14.9 years (13–19 years)	5 M 21 F	NR	Adolescents with self-reported insomnia: HSDQ criteria	N/A
de Bruin et al. (2015a) (Netherlands)	2-arm parallel group (WL) No FU	32	15.9 years (13–19 years)	6 M 26 F	NR	Adolescents with self-reported insomnia: DSM-5 criteria, HSDQ	N/A
de Bruin et al. (2015b, 2018)^†^ (Netherlands)	3-arm parallel group (WL) 2-, 6-, 12-month FU	116	15.6 years (12–19 years)	29 M 87 F	93 The Netherlands 10 Other 13 Missing (Parent country of birth)	Adolescents with insomnia: DSM-IV / DSM-5 criteria	N/A
Corkum et al. (2016) (Canada)	2-arm parallel group (WL) 6-month FU	61 (22 ADHD, 39 TD; 61 parents)	108 months (5–12 years)	28 M 33 F	53 Caucasian 8 Other	Parents of children with trouble falling asleep with/without bedtime resistance: SOL >25min, ≥3 times/week (based on DSM-IV criteria for primary insomnia)	Subsample with ADHD diagnosis
Roberts et al. (2019) (USA)	2-arm parallel (F2F) 8-week FU	23 (23 parents)	7 years and 11 months (4–12 years)	15 M 8 F	NR	Children with sleep problems (parent reported)	Sample with ASD diagnosis; comorbidities: 47.8% with ADHD diagnosis, 30.4% with anxiety (parent reported)
Werner-Seidler et al. (2023) (Australia)	2-arm parallel group (active) 14-week FU	264	14.71 years (12.05 – 16.92)	72 M 189 F 4 Other	248 born in Australia	≥10 on the Insomnia Severity Index	Moderately elevated levels of depression and anxiety.

^#^Initial pre-post outcomes are reported in Mindell et al. (2011a), while 1-year follow-up outcomes are reported subsequently in Mindell et al. (2011b). ^†^Initial pre-post and 2-month follow-up outcomes are reported in de Bruin et al. (2015b), while 6- and 12-month follow-up outcomes are reported subsequently in de Bruin et al. (2018). ADHD, Attention-Deficit Hyperactivity Disorder; ASD, Autism Spectrum Disorder; DSM, Diagnostic and Statistical Manual of Mental Disorders; F2F, face-to-face; FU, Follow-up; HSDQ, Holland Sleep Disorders Questionnaire (Kerkhof et al., 2012); NR, not reported; TDC, typically developing; SOL, sleep onset latency; TAU, treatment as usual; WL, Waiting List.

Overall, the studies included 786 participants, with the number of participants in individual studies ranging from 26 to 264. The mean age of participants ranged from 19.3 months to 16.9 years. Studies examined effectiveness of treatments in adolescents (*n* = 4), school aged children (*n* = 2) and infants/toddlers (*n* = 1). Two of the seven studies examined effectiveness of treatments for insomnia/symptoms of insomnia in children with neurodevelopmental disorders, with one study including children with attention deficit hyperactivity disorder (ADHD; Corkum et al., 2016) alongside neurotypical children, and another study including only children with autism spectrum disorder (ASD; Roberts et al., 2019).

Inclusion criteria. In 6 studies that included school aged children/adolescents, participants had to meet Diagnostic and Statistical Manual of Mental Disorders, Fifth Edition/Fourth Edition (DSM-5/DSM-IV; American Psychiatric Association, 2000, 2013) or Holland Sleep Disorders Questionnaire (HSDQ; Kerkhof et al., 2012) criteria for insomnia/showed insomnia symptoms; or scored ≥10 on the Insomnia Severity Index (ISI, Bastien et al., 2001). In a study that enrolled infants and toddlers, children were included if their mothers endorsed (i) sleep problems (“small” to “serious”) and (ii) bedtime difficulties (“somewhat difficult” to “very difficult”). In a study that enrolled children with ASD, children were included when parents reported that their children had current sleep problems.

### 3.2 Design

Of the 7 studies, 2 studies used a 3-arm design (remote, face-to-face, and waitlisted/no treatment) and 5 studies used a 2-arm design (remote vs face-to-face (*n* = 2); remote vs waitlisted (*n* = 2); both remote: CBT-I vs active control). All studies evaluated outcomes post-treatment. Five studies included a follow-up at 2 – 12 months post-treatment, with 1 of these 5 studies including multiple follow-ups, at 2-, 6- and 12-months post-treatment.

### 3.3 Treatment characteristics

Characteristics of treatment used in studies are provided in Table 2. Four different treatments were used.

**Table 2:** Intervention characteristics and outcome measures.

**Study**	**Intervention**	**Outcome Measures**			
**References (Country)**	**T: Sleep intervention(s) Availability of Intervention C: Control condition(s)**	**Sleep: subjective**	**Sleep: objective**	**Cognitive and academic**	**Mood/behavior/quality of life/functional**
Mindell et al. (2011a,b) (USA)	T_1_: Customized Sleep Profile Delivery: Remote (automated) Platform: Mobile application or website Sessions: N/A (automated) Duration: 2 weeks Sleep components: -Psychoeducation -Bedtime routine -Stimulus control -Extinction of negative sleep associations Availability: only one aspect of the program is readily available: bedtime routine (free of charge) via Johnson and Johnson's website T_2_: Customized sleep profile (as in T_1_) + 3-step bedtime routine: bath, lotion massage and quiet activity Delivery: remote (automated) C: No treatment (bedtime routine as usual)	Child BISQ: SOL, WASO duration and frequency, TST, SSQ Mother PSQI: SOL, WASO duration and frequency, TST, SE, SSQ	NR	NR	Child BISQ: mood in the morning Mother POMS: maternal mood
de Bruin et al. (2014) (Netherlands)	T: sleeping smart (internet) Delivery: remote (automated, with option to send questions and comments to therapists) + a 15 min chat session with a therapist after the second session Platform: website Sessions: 6 Duration: 6 weeks Sleep components: -Psychoeducation -Sleep hygiene -Sleep restriction -Cognitive restructuring -Relaxation Availability: NR C: Sleeping Smart (Group) Delivery: Face to face	Sleep Diaries: SE, TST, SOL, WASO duration, TIB HSDQ: insomnia symptoms CSRQ: chronic sleep reduction	Actigraphy: SE, TST, SOL, WASO duration, TIB	NR	NR
de Bruin et al. (2015a) (Netherlands)	T: Sleeping smart Delivery: Remote (automated, with option to send questions and comments to therapists) + a 15 min chat session with a therapist after the second session Platform: website Sessions: 6 Duration: 6 weeks Sleep components: -Psychoeducation -Sleep hygiene -Sleep restriction -Cognitive restructuring -Relaxation Availability: NR C: WL	Sleep Diaries: SE, SOL, WASO, TST, TIB, SSQ HSDQ: insomnia symptoms CSRQ: chronic sleep reduction Clinically significant insomnia	Actigraphy: SE, SOL, WASO, TST, TIB, FI	ANT: simple reaction time, visuospatial processing, selective attention and working memory, response inhibition and set shifting, visuospatial working memory (5 subtests) PVT: sustained attention AVLT: declarative memory Letter Fluency: language, executive function Category Fluency: semantic processing	NR
de Bruin et al. (2015b, 2018) (Netherlands)	T_1_: Sleeping smart (Internet) Delivery: remote (automated, with option to send questions and comments to therapists) + a 15 min chat session with a therapist after the second session Platform: website Sessions: 6, optional 15-min online chat Duration: 6 weeks Sleep components: -Psychoeducation -Sleep hygiene -Sleep restriction -Cognitive restructuring -Relaxation Availability: NR T_2_: Sleeping smart (Group) Delivery: Face to face C: WL	Sleep diaries: SE, TST, SOL, WASO duration, TIB, SSQ HSDQ: insomnia symptoms CSRQ: chronic sleep reduction	Actigraphy: SE, TST, SOL, WASO duration, TIB	NR	Child YSR: psychopathology symptoms^#^
Corkum et al. (2016) (Canada)	T: Better Nights Better Days Platform: website Sessions: 5 × 30–45 min telephone support Duration: 5 weeks Sleep components: -Bedtime routines -Sleep hygiene -FBRC-PR Availability: NR C: WL	CSHQ: sleep disturbances	Actigraphy: SOL, TST	NR	Child CBCL: child behavior/daytime functioning
Roberts et al. (2019) (USA)	T: parent education for children with autism (online) Delivery: Remote Platform: website Sessions: 2 × 2-h podcast sessions, optional online blogging and emails to instructor Duration: 1–2 weeks Sleep components: -Psychoeducation and general sleep strategies -Bedtime routines -Reinforcements -Self soothing Availability: C: Parent education for children with autism Delivery: Face to face Sessions: 2 × 2-hour interactive workshops	CSHQ: sleep problems and insomnia symptoms	Actigraphy: SOL, TST, SE, WASO duration, WASO frequency	NR	Parents PedsQL^TM^: parent HR-QoL FAS: parents' fatigue
Werner-Seidler et al. (2023) (Australia)	T: Sleep ninja Platform: smartphone app Sessions: 6 × 5–10 min Duration: 5 weeks Sleep components: -Psychoeducation -Stimulus control -Sleep hygiene -Cognitive therapy -Summary and relapse prevention -FBRC-PR Availability: Google Play Delivery: Remote (smartphone app) C: WL	ISI: insomnia symptoms PSQI: sleep quality SRBQ: sleep behaviors ESS: daytime sleepiness DBAS: attitudes and beliefs about sleep PSAS: arousal before sleep	NR	NR	PHQ-A: depressive symptoms GAD-7: anxiety FFS: fatigue SWEMWBS: well-being

^#^Reported exclusively in de Bruin et al. (2018). ANT, Amsterdam Neuropsychological Tasks (de Sonneville, 1999); AVLT, Auditory Verbal Learning Test (Lezak et al., 2004); BISQ, Brief Infant Sleep Questionnaire (Sadeh, 2004); C, control condition; CBCL, Child Behavior Checklist (Achenbach and Rescorla, 2001); CSHQ, Children's Sleep Habits Questionnaire (Owens et al., 2000); CSRQ, Chronic Sleep Reduction Questionnaire (Meijer, 2008); DBAS, Dysfunctional Beliefs and Attitudes about Sleep Scale for Children (Blunden et al., 2013); ESS, Epworth Sleepiness Scale for Children and Adolescents (Janssen et al., 2017); FAS, Fatigue Assessment Scale (Michielsen et al., 2003); FFS, Flinders Fatigue Scale (Gradisar et al., 2007); FI, fragmentation index; F2F, face-to-face; GAD-7, Generalized Anxiety Disorder 7 (Spitzer et al., 2006; Tiirikainen et al., 2019); HR-QoL, health-related quality of life; ISI, Insomnia Severity Index (Bastien et al., 2001); NR, not reported; PedsQL^TM^, Pediatric Quality of Life Inventory TM (World Health Organisation, 1995); PHQ-A, Patient Health Questionnaire- Adolescent Version (Johnson et al., 2002); POMS, Profile of Mood States (Shacham, 1983); PSAS, Pre-Sleep Arousal Scale (Nicassio et al., 1985); PSQI, Pittsburgh Sleep Quality Index (Buysse et al., 1989; de la Vega et al., 2015); PVT, Psychomotor Vigilance Task (Dinges and Powell, 1985); SE, sleep efficiency; SOL, sleep onset latency; SRBQ, Sleep-Related Behaviors Questionnaire (Ree and Harvey, 2004); SSQ, subjective sleep quality; SWEMWBS, Short Warwick-Edinburgh Mental Wellbeing Scale (Tennant et al., 2007); T, treatment; TIB, time in bed; TST, total sleep time; WASO, wakefulness after sleep onset; WL, Waiting List; YSR, Youth Self-Report (Achenbach and Rescorla, 2001).

Sleep Smart program, based on a CBT-I, was used in studies conducted by de Bruin et al. (2014, 2015a,b, 2018) (*n* = 3). The program was developed for and delivered to adolescents. Parents received a short booklet about the program and were informed that their child could participate in the program independently. Nevertheless, parents could offer support with implementation of certain aspects of the program. The program contained the following components: sleep hygiene and sleep education, sleep restriction, stimulus control, cognitive therapy, and relaxation. Participants logged into a personalized website and worked through information and exercises presented in a fixed order. Adolescents completed a short questionnaire a day before the session. Based on the answers provided on the questionnaire and sleep diaries, participants were provided with written personalized feedback from a sleep therapist. On completion of the second internet therapy session, participants were offered a 15 min online chat with therapist to boost their motivation early in the treatment. The Sleep Smart program was delivered once per week, at a specific time and day of the week, over 6 weeks, followed by a booster session delivered 2 months after completion of the 6th treatment session.

Better Nights Better Days program, used by Corkum et al. (2016), is a distance intervention for school aged children (5–10 years) with insomnia. Parents were provided with a written manual. The program also included an online component, which was completed by parents autonomously. In addition, parents attended a weekly 30–45 min telephone consultation with a sleep coach, during which weekly modules that were completed online were discussed. The content of the program was standardized, and included the following components: sleep hygiene, sleep physiology, and how to handle early morning awakenings. The program also included faded bedtime with response and cost reinforcement. Telephone sessions were used to tailor the program content to each family. The program was delivered over 5 weeks.

The Sleep Ninja, employed in a study by Werner-Seidler et al. (2023), is a smartphone app for adolescents. The intervention was developed in collaboration with adolescents. It contains a CBT-I program that is delivered in 6 short (5 min−10 min) training sessions. The program includes the following components: psychoeducation, stimulus control, sleep hygiene, identification and planning of high-risk situations/diversion from the sleep routine, cognitive therapy, summary of the program and relapse prevention. The program is automatized and gamified; with participants progressing from one level to the next and gaining “belts,” until they reach the highest level and “black belt” for sleep. The program also includes “Sleep Tips” and “Get Help Now,” with later containing crisis support lines (as mood comorbidities are common in this population). The program does not include any contact with a therapist.

Educational sessions were developed for parents of school aged children with autism by Roberts et al. (2019). The sessions were based on the Solving Sleep Problems in Children with Autism (Katz and Malow, 2014). Materials used in the sessions involved slides and 4 informational videos demonstrating how to use parenting behavioral strategies to promote sleep, such as visual schedules, sleeping alone, bed-time pass (a token that a child could use if checking in with parents during the night), and staying in bed. In addition, parents were provided with the Sleep Toolkit (Strategies to Improve Sleep in Children with Autism Spectrum Disorders) from the Autism Speak website and “… the parent teaching checklist (PTC) to monitor the sleep setting, bedtime routine, regularity of the schedule, any interventions used to facilitate self-soothing and independent sleep, and daily activities. Space was provided on the PTC for parents to list additional strategies or thoughts” (p. 1413). The educational sessions were presented in 2 workshops (2-h podcast session each), 1 to 2 weeks apart. The sessions included (i) psychoeducation about sleep (bedtime habits, and environment), (ii) behavioral parenting strategies. In addition, online blogging and e-mails to the instructor were offered to parents, if needed.

An information prescription intervention for children with behavioral sleep disturbances aged 6–36 months was evaluated by Mindell et al. (2011a,b). Parents completed an extended version of the Brief Infant Sleep Questionnaire (BISQ, Sadeh, 2004) online. The extended BISQ provided information on child's sleep, namely: TST, SOL and WASO (number and duration) at night. The Customized Sleep Profile algorithm compared sleep parameters of an individual child on the extended BISQ to age-based norms, determined whether a child was “an excellent, good or disrupted sleeper” and provided an individually tailored advice to parents as to how to facilitate their children's sleep at night. The intervention was fully automatized. A randomly selected subgroup of participants was asked to concurrently implement the automatized sleep intervention and a general 3-step bedtime routine that was previously found to significantly improve sleep (Mindell et al., 2009).

#### 3.3.1 Outcome measurement

Primary outcomes (see Table 2). Subjective and objective measures of child sleep were employed in 7 and 5 of 7 studies, respectively. Subjective measures included different self or parent completed questionnaires in 7 studies and sleep diaries in 3 studies. Sleep diaries were used in combination with questionnaires (*n* = 2) or alone (*n* = 1). Actigraphy was the only objective measure of sleep used in the studies. No study used polysomnography.

Questionnaires provided total scores and subscale scores that measured various aspects of sleep disturbance, as perceived by parents/participants. Specific sleep parameters were obtained from actigraphy in 5 out of 7 RCTs, and sleep diaries and different questionnaires in 6 out of 7 RCTs (see Tables 2, 3), with total sleep time (TST) and sleep onset latency (SOL) reported in in all 6 studies, WASO in 5 and SE in 3 studies.

**Table 3:** Specific sleep parameters: (i) significant improvements (I), no change (0), significant worsening (W), and (ii) means, standard deviations, from pre- to post-intervention and on follow-up.

**References**	**Type of measure**	**Specific sleep parameter**							
		**TST (hr)**		**SOL (min)**		**WASO (min)**		**SE (%)**	
		**Pre-post**	**Follow-up**	**Pre-post**	**Follow-up**	**Pre-post**	**Follow-up**	**Pre-post**	**Follow-up**
Mindell et al. (2011a,b)	Questionnaire	I (Pre: 9.49, 1.53; Post: 10.16, 1.22)^a^	M (10.13, 1.22)	I (Pre: 25.49, 16.33; Post: 14.22, 11.83)	M (15.69, 11.97)	I (Pre: 46.41, 42.87; Post: 22.81, 32.35)	M (15.6, 34.2)		
de Bruin et al. (2014)	Sleep Diary	I (Pre: 7.7, 1.0; Post: 8.0, 0.7)	M (8.0, 0.6)	I (Pre: 44.3, 42.2; Post: 21.9, 10.9)	M (16.2, 8.0)	I (Pre: 8.3, 14.2; Post: 1.0, 2.8)	M (3.3, 8.0)	I (Pre: 87.4, 8.9; Post: 92.8, 2.3)	M (94.4, 3.0)
	Actigraphy	0 (Pre: 7.3, 0.9; Post: 7.5, 0.9)	0 (6.9, 0.5)	I (Pre: 15.9, 15.3; Post: 14.4, 17.3)	M (13.1, 10.9)	0 (Pre: 79.7, 30.6; Post: 82.8, 35.2)	0 (63.2, 24.8)	I (Pre: 81.4, 5.4; Post: 81.8, 6.3)	M (84.1, 5.1)
de Bruin et al. (2015a)	Sleep Diary	0 (Pre: 7.2, 1.3; Post: 7.6, 1.2)^b^		I (Post: 31, 20; Pre: 52, 38)		0 (Post: 2, 5; Pre: 14, 27)		I (Post: 90.4, 4.9; Pre: 82.1, 10)	
	Actigraphy	0 (Pre: 6.68, 0.93; Post: 6.73, 0.65)		0 (Post: 16, 14; Pre: 24, 17)		0 (Post: 70, 25; Pre: 81, 35)		I (Pre: 78.3, 7.4; Post: 82.1, 5.8)	
de Bruin et al. (2015b, 2018)	Sleep diary	0 (Pre: 7.67, 1.5; Post: 7.90, 1.22)	0 (8.00, 1.23)	I (Post: 30, 27; Pre: 49, 44)	M (32, 25)	0 (Post: 5, 12; Pre: 12, 25)	0 (6, 14)	I (Pre: 84.59, 10.02; Post: 90.61, 7.24)	M (90.30, 6.88)
	Actigraphy	I (Pre: 6.98, 1.25; Post: 7.27, 1.10)	M (7.12, 0.95)	I (Post: 20, 15; Pre: 39, 24)	M (18, 15)	0 (Post: 73, 29; Pre: 74, 29)	0 (72, 25)	I (Pre: 76.70, 6.44; Post: 82.55, 5.25)	M (82.72, 5.06)
Corkum et al. (2016)	Questionnaire^c^	I (Pre: 5.68, 1.66; Post: 4.26, 1.59)	M (4.48, 1.77)	I (Pre: 2.55, 0.72; Post: 1.45, 0.57)	M (1.68, 0.79)				
	Actigraphy	0 (Pre: 9.11, 0.63; Post: 8.93, 0.82)	0 (9.08, 0.71)	W (Pre: 46.04, 20.81; Post: 52.61, 23.25)	I (36.24, 18.02)				
Roberts et al. (2019)	Questionnaire^c^	I (Pre: 6.20, 2.44; Post: 4.40, 1.90)	M^d^	I (Pre: 2.10, 0.74; Post: 1.40, 0.70)	M	I (Pre: 6.20, 1.55; Post: 4.10, 1.45)	M		
	Actigraphy	0 (Pre: 8.44, 0.66; Post: 8.49, 0.66)		0 (Pre: 9.87, 8.18; Post: 16.75, 9.93)		W (Pre: 42.46, 14.13; Post: 55.44, 14.78)		0 (Pre: 86.17, 5.62; Post: 84.96, 2.30)	
Werner-Seidler et al. (2023)									
Summary of changes		I, *n =* 5 0, *n =* 6 W, *n =* 0	M, *n =* 5 0, *n =* 3 W, *n =* 0	I, *n =* 8 0, *n =* 2 W, *n =* 1	M = 7 0, NR I, *n =* 1	I, *n =* 3 0, *n =* 5 W, *n =* 1	M, *n =* 3 0, *n =* 3 W, NR	I, *n =* 6 0, *n =* 1 W, *n =* 0	M, *n =* 4 0, NR W, *n =* 0

^*a*^Post scores taken from week 3. Scores are for the internet intervention only (not internet + routine); ^*b*^Means and SDs taken from school days (and not free days); ^*c*^Sleep outcomes were assessed using the CSHQ (Owens et al., 2000) where lower scores indicate improvement instead of time-based indices; ^*d*^follow-up means and standard deviations were not reported. NR, not reported.

Secondary outcomes were assessed in 6 studies. Changes in children's mood or behavior was assessed with different parent or self-completed standardized instruments. Fatigue and wellbeing were evaluated in one study. Only 1 of the 6 studies assessed changes in children's cognition (attention, reaction time, episodic memory, language, visuo-spatial processing, and executive skills (inhibition, shifting, working memory). No study examined children's quality of life or academic skills. Two studies reported on changes in parental wellbeing: mood, quality of life and fatigue.

### 3.4 Treatment outcomes of remotely delivered interventions

Frequency of significant changes and descriptive statistics of main sleep parameters from pre- to post-treatment and follow-up are provided in Table 3. Table 4 contains details for primary and secondary outcomes of remotely delivered interventions.

**Table 4:** Intervention outcomes.

**References (Country)**	**Time of assessments**	**Sleep outcomes**		**Functional outcomes**	
		**Subjective**	**Objective**	**Cognitive and academic**	**Mood/behavior/functional**
Mindell et al. (2011a,b) (USA)	Pre-post	**Child BISQ:** SOL: Week 2 < Baseline^#†^; Week 3 < Baseline ^#†^ WASO duration: Week 2 < Baseline^#†^; Week 3 < Baseline^#†^ WASO frequency: Week 2 < Baseline^#†^; Week 3 < Baseline^#†^ TST: Week 2 > Baseline^#†^; Week 3 > Baseline^#†^ SSQ: Week 2 > Baseline^#†^; Week 3 > Baseline^#†^ **Mother PSQI:** SOL: Week 2 < Baseline^#†^; Week 3 < Baseline^#†^ WASO duration: Week 2 < Baseline^#†^; Week 3 < Baseline^#†^ WASO frequency: Week 2 < Baseline^#†^; Week 3 < Baseline^#†^ TST: Week 2 < Baseline^#†^; Week 3 < Baseline^#†^ SSQ: Week 2 < Baseline^#†^; Week 3 < Baseline^#†^ SE: Week 3 > Baseline^*#†*^	NR	NR	**Child BISQ:** Mood in morning: Week 2 > Baseline^#†^; Week 3 > Baseline^#†^ **Mother POMS:** *Tension:* Week 2 < Baseline^#†^; Week 3 < Baseline^#†^ *Depression:* Week 2 < Baseline^#†^; Week 3 < Baseline^#†^ *Anger:* Week 2 < Baseline^†^; Week 3 < Baseline^†^ *Fatigue:* Week 2 < Baseline^#†^; Week 3 < Baseline^#†^ *Vigor:* Week 2 < Baseline^†^; Week 3 < Baseline^†^ *Confusion:* Week 2 < Baseline^#†^; Week 3 < Baseline^*#†*^
	Follow up	**Child BISQ:** SOL: 1-year FU < Baseline^#^; 1-year FU = Week 3^#†^ WASO duration: 1-year FU < Baseline^#†^; 1-year FU = Week 3^#†^ WASO frequency: 1-year FU < Baseline^#†^; 1-year FU = Week 3^#†^ TST: 1-year FU = Week 3^#†^ SSQ: NR **Mother PSQI:** SSQ: 1-year FU = Week 3^*#†*^	NR	NR	**Child BISQ:** Mood in morning: NR **Mother POMS:** NR
de Bruin et al. (2014) (Netherlands)	Pre-post	**Sleep diaries:** SE: Post-treatment > Baseline TST: Post-treatment > Baseline SOL: Post-treatment < Baseline WASO duration: Post-treatment < Baseline TIB: Post-treatment > Baseline **HSDQ:** Insomnia: Post-treatment < Baseline **CSRQ:** Total: Post-treatment < Baseline Shortage of sleep: Post-treatment < Baseline Loss of energy: Post-treatment < Baseline Sleepiness: Post-treatment < Baseline	**Actigraphy:** SE: Post-treatment > Baseline TST: n/s SOL: Post-treatment < Baseline WASO duration: n/s TIB: n/s	NR	**Child CSRQ** Irritation: n/s
	Follow up	**Sleep Diaries:** SE: 2-month FU = Post-treatment TST: 2-month FU = Post-treatment SOL: 2-month FU = Post-treatment WASO duration: 2-month FU = Post-treatment TIB: 2-month FU = Post-treatment **HSDQ:** Insomnia: 2-month FU < Post-treatment **CSRQ:** Total: 2-month FU < Post-treatment Shortage of sleep: 2-month FU = Post-treatment Loss of energy: 2-month FU = Post-treatment Sleepiness: 2-month FU = Post-treatment	**Actigraphy:** SE: 2-month FU = Post-treatment TST: n/s SOL: 2-month FU = Post-treatment WASO duration: n/s TIB: n/s	NR	**Child CSQR** Irritation: 2-month FU < Post-treatment
de Bruin et al. (2015a) (Netherlands)	Pre-post	**Sleep Diaries** SE: Post-treatment > Baseline TST: Post-treatment > Baseline SOL: Post-treatment < Baseline WASO duration: Post-treatment < Baseline TIB: n/s SSQ: Post-treatment > Baseline **HSDQ:** Insomnia: Post-treatment < Baseline **CSRQ:** Total: Post-treatment < Baseline Shortage of sleep: Post-treatment < Baseline Loss of energy: Post-treatment < Baseline Sleepiness: n/s	**Actigraphy** SE: Post-treatment > Baseline TST: n/s SOL: n/s WASO duration: n/s TIB: n/s FI: n/s	**ANT:** Simple reaction time: n/s Visuospatial processing: *Reaction Time:* Post-treatment > Baseline *Proportion Correct:* Post-treatment > Baseline *Efficiency:* Post-treatment > Baseline Selective attention and working memory (phonological):	**Child CSRQ** Irritation: Post-treatment < Baseline
				*Efficiency:* Post-treatment > Baseline Response inhibition and set shifting: n/s^*^ Visuospatial working memory: n/s **PVT:** n/s^*^AVLT: n/s^*^Letter Fluency: n/s^*^Category Fluency: n/s^*^^*^no significant interaction between condition and time despite significant main effect of time **Correlations with Post-treatment sleep outcomes on school nights:** Visuospatial processing: improvements associated with improvements of WASO duration, SSQ on *Sleep Diaries*; loss of energy, sleepiness, and total score on *CSRQ* Selective attention and working memory: improvements associated with WASO duration on *Sleep Diaries*; shortness of sleep on *CSRQ* Sustained attention: improvements associated with increase in WASO duration on *Sleep Diariess*	
	Follow up	NR	NR	NR	NR
de Bruin et al. (2015b, 2018) (Netherlands)	Pre-post	**Sleep diaries** SE: Post-treatment > Baseline TST: n/s SOL: Post-treatment < Baseline WASO duration: n/s TIB: n/s SSQ: Post-treatment > Baseline **HSDQ:** Insomnia: Post-treatment < Baseline **CSRQ:** Total: Post-treatment < Baseline	**Actigraphy** SE: Post-treatment > Baseline TST: Post-treatment > Baseline SOL: Post-treatment < Baseline WASO duration: n/s TIB: n/s	NR	**Child YSR:** Affective: Post-treatment < Baseline Anxiety: Post-treatment < Baseline Somatic: Post-treatment < Baseline ADHD: Post-treatment < Baseline Oppositional defiant: n/s Conduct: n/s
	Follow up	**Sleep diaries:** SE: 2-month FU= Post-treatment; 6-, 12-month FU = 2-month FU TST: n/s SOL: 2-month FU = Post-treatment; 6-, 12-month FU = 2-month FU WASO duration: n/s^*^ TIB: n/s^*^ SSQ: 2-month FU = Post-treatment^*^HSDQ: Insomnia: 2-month FU = Post-treatment; 6-, 12-month FU = 2-month FU **CSRQ:** Total: 2-month FU = Post-treatment^*^	**Actigraphy:** SE: 2-month = Post-treatment; 6-, 12-month FU = 2-month FU TST: 2-month FU = Post-treatment; 6-, 12-month FU = 2-month FU SOL: 2-month FU = Post-treatment; 6-, 12-month FU = 2-month FU WASO duration: n/s^*^ TIB: n/s^*^	NR	**Child YSR:** Affective: 2-month FU < Baseline; 2-month FU = Post-treatment; 6-month, 12-month FU < 2-month FU Anxiety: 2-month FU < Baseline; 2-month FU < Baseline; 2-month FU = Post-treatment; 6-, 12-month FU = 2-month FU Somatic: 2-month FU < Baseline; 2-month FU = Post-treatment; 6-, 12-month FU = 2-month FU ADHD: 2-month FU < Baseline; 2-month FU < Post-treatment; 6-, 12-month FU = 2-month FU Oppositional defiant: 2-month FU < Baseline Conduct: n/s
Corkum et al. (2016) (Canada)	Pre-post	**CSHQ:** Total: Post-treatment < Baseline Bedtime resistance: Post-treatment < Baseline SOL: Post-treatment < Baseline TST: Post-treatment > Baseline	**Actigraphy** SOL: Post-treatment > Baseline TST: n/s	NR	**Child CBCL:** Internalizing (t-score): Post-treatment < Baseline Externalizing (t-score): Post-treatment < Baseline
	Follow up	**CSHQ:** Total: 6-month FU = Post-treatment Bedtime resistance: 6-month FU = Post-treatment SOL: 6-month FU = Post-treatment Sleep duration: 6-month FU = Post-treatment	**Actigraphy** SOL: 6-month FU < Baseline TST: n/s	NR	**Child CBCL:** Internalizing (t-score): 6-month FU = Post-treatment Externalizing (t-score): 6-month = Post-treatment
Roberts et al. (2019) (USA)	Pre-post^a^	**CSHQ** Total: Post-treatment < Baseline Bedtime Resistance: Post-treatment < Baseline SOL: Post-treatment < Baseline TST: Post-treatment > Baseline Parasomnias: n/s WASO: Post-treatment < Baseline Breathing: n/s Sleepiness: n/s	**Actigraphy:** SOL: Post-treatment > Baseline TST: n/s SE: n/s WASO duration: Post-treatment > Baseline WASO frequency: n/s	NR	**Children CSHQ** Sleep anxiety: Post-treatment < Baseline **Parents PedsQL**^**TM**^**:** Post-treatment > Baseline **FAS:** Post-treatment < Baseline
	Follow up	**CSHQ** Total: 8-week FU = Post-treatment Bedtime resistance: 8-week FU = Post-treatment Sleep onset delay: 8-week FU = Post-treatment Sleep duration: 8-week FU = Post-treatment Parasomnias: n/s Wakings: 8-week FU = Post-treatment Breathing: n/s Sleepiness: n/s	**Actigraphy:** NR	NR	**Children CSHQ** Sleep anxiety: 8-week FU = Post-treatment **Parents PedsQL**^**TM**^**:** 8-week FU > Post-treatment **FAS:** 8-week FU < Post-treatment
Werner-Seidler et al. (2023) (Australia)	Pre-post	**ISI**: Post-treatment < Baseline **PQSI:** Post-treatment < Baseline **SRBQ**: n/s **ESS:** n/s **DBAS**: Post-treatment < Baseline **PSAS:** Post-treatment < Baseline	**Actigraphy**: N/R	NR	**PHQ-A**: Post-treatment < Baseline **GAD-7:** Post-treatment < Baseline **FFS**: n/s **SWEMWBS**: n/s
	Follow-up	**ISI**: Follow-up < Baseline **PQSI**: Follow-up < Baseline **SRBQ**: n/s **ESS**: n/s **DBAS**: Follow-up < Baseline **PSAS**: n/s	**Actigraphy:** N/R	NR	**PHQ-A**: n/s **GAD-7**: n/s **FFS**: n/s **SWEMWBS**: n/s

^*a*^The first assessment was completed 4 weeks Post-treatment (Roberts et al., 2019). ^#^Results refer to the T_1_ intervention group (Customized Sleep Profile only, Mindell et al., 2011a,b). ^†^Results refer to the T_2_ intervention group (Customized Sleep Profile and 3-step Bedtime Routine, Mindell et al., 2011a,b). ^*^6-, 12-month follow-up data were not available for these outcomes (de Bruin et al., 2018). ANT, Amsterdam Neuropsychological Tasks (de Sonneville, 1999); AVLT, Auditory Verbal Learning Test (Lezak et al., 2004); BISQ, Brief Infant Sleep Questionnaire (Sadeh, 2004); C, control condition; CBCL, Child Behavior Checklist (Achenbach and Rescorla, 2001); CSHQ, Children's Sleep Habits Questionnaire (Owens et al., 2000); CSRQ, Chronic Sleep Reduction Questionnaire (Meijer, 2008); DBAS, Dysfunctional Beliefs and Attitudes about Sleep Scale for Children (Blunden et al., 2013); ESS, Epworth Sleepiness Scale for Children and Adolescents (Janssen et al., 2017); FAS, Fatigue Assessment Scale (Michielsen et al., 2003); FFS, Flinders Fatigue Scale (Gradisar et al., 2007); FI, fragmentation index; F2F, face-to-face; GAD-7, Generalized Anxiety Disorder 7 (Spitzer et al., 2006; Tiirikainen et al., 2019); HR-QoL, health-related quality of life; ISI, Insomnia Severity Index (Bastien et al., 2001); NR, not reported; PedsQL^*TM*^, Pediatric Quality of Life Inventory TM (World Health Organisation, 1995); PHQ-A, Patient Health Questionnaire- Adolescent Version (Johnson et al., 2002); POMS, Profile of Mood States (Shacham, 1983); PSAS, Pre-Sleep Arousal Scale (Nicassio et al., 1985); PSQI, Pittsburgh Sleep Quality Index (Buysse et al., 1989; de la Vega et al., 2015); PVT, Psychomotor Vigilance Task (Dinges and Powell, 1985); SE, sleep efficiency; SOL, sleep onset latency; SRBQ, Sleep-Related Behaviors Questionnaire (Ree and Harvey, 2004); SSQ, subjective sleep quality; SWEMWBS, Short Warwick-Edinburgh Mental Wellbeing Scale (Tennant et al., 2007); T, treatment; TIB, time in bed; TST, total sleep time; WASO, wakefulness after sleep onset; WL, Waiting List; YSR, Youth Self-Report (Achenbach and Rescorla, 2001).

#### 3.4.1 Primary outcomes

Significant improvements in child sleep were found across studies on subjective (questionnaires and sleep diaries) and objective (actigraphy) measures. All studies that that used questionnaires and reported total scores (*n* = 6/6) found significant improvements from pre- to post-treatment. In a study that used multiple follow-ups, gains in total scores were maintained over time, and were still evident up to 6 months post-treatment. Significant improvements were also found on various specific aspects of sleep measured by questionnaires (see Table 4 for details).

Across studies and instruments (i.e., questionnaires, sleep diaries, actigraphy) changes in specific sleep parameters were reported 44 times pre- to post-treatment and 26 times on the first follow-up (Table 3). Of the 44 measurements, significant improvements, no change, and significant worsening, were recorded in 28, 14, and 2 times pre- to post-treatment. Consistency of significant changes in specific sleep parameters, however, was varied:

(i) SE was reported and improved in all 3 studies from pre- to post-intervention, irrespective of the measures used (sleep diary, actigraphy), and the improvements were maintained on follow-ups (in 2 of 2 studies that conducted follow-ups).
(ii) SOL improved in all 6 studies on questionnaires/sleep diaries from pre- to post-intervention and remained improved on follow-ups. On actigraphy, however, SOL improved, worsened, and did not change significantly in 2, 2, and 1 study, respectively. On follow-ups, actigraphy recorded improvements were maintained, and the pre-post decline turned to an improvement relative to baseline.
(iii) TST improved in 5 of the 6 studies; the improvements were found on questionnaires/sleep diary in 4 studies, and on actigraphy in 1 study.
(iv) Finally, WASO improved in 3 of the 5 studies pre- to post-intervention, as measured by questionnaires/sleep diary and worsened in 1 study, measured by actigraphy. On follow-ups, improvements were maintained. Actigraphy was not used on the follow-up to determine whether worsening persisted or changed.

##### 3.4.1.1 Remotely delivered interventions compered to face-to-face delivered interventions

Three studies examined whether sleep outcomes were differentially impacted by the modality of treatment delivery: remote or face-to-face. de Bruin et al. (2015b) compared sleep outcomes of 3 conditions: waitlisted, internet delivered treatment and face-to-face delivered treatment. Sleep significantly improved in both treatment conditions relative to the waitlist condition. Nevertheless, the study revealed an interaction on: SE (actigraphy and sleep logs), SOL (actigraphy) and WASO duration (sleep diaries), with less improvements following the internet delivered treatment. TST did not change. Two studies (de Bruin et al., 2014; Roberts et al., 2019) compared sleep outcomes of two treatment conditions: face-to-face and remote. de Bruin et al. (2014) found significant improvements in SE, SOL and WASO for both treatment conditions. Nevertheless, the remotely delivered treatment resulted in less improvement in (i) SE (actigraphy and sleep diaries), (ii) SOL and TIB (actigraphy), and WASO (sleep diaries). In a study by Roberts et al. (2019) sleep improved following face-to-face, and podcast delivered treatment: overall score (questionnaire), SOL (questionnaire and actigraphy) and bedtime resistance (questionnaire). An interaction was found for WASO on both questionnaire and actigraphy, albite in different directions. On the questionnaire improvement was found in the podcast but not face-to-face condition. On the actigraphy, WASO duration increased (worsened) in the podcast group and remained unchanged in the face-to-face group.

#### 3.4.2 Secondary outcomes

##### 3.4.2.1 Children

Five studies examined changes in mood and/or behavior from pre- to post-treatment in children. In infants and toddlers, there was an improvement of mood in the morning on completion of treatment. In school aged children, caregiver completed questionnaires showed significant improvements in mood and behavior (Externalizing score on the CBCL) posttreatment and on follow-up. In adolescents, self-reported questionnaires showed improvements in mood (Affective and Anxiety scales of the YSR), but not behavior (Oppositional and Conduct scales of the YSR) from pre- to post-treatment. In addition, irritability decreased in two studies; in one study, on completion of treatment, and in another on follow-up (but not on completion of treatment). Improvements in mood were maintained at 2-, 6-, and 12-months post-treatment, with further improvement noticed in anxiety at 12 months (*n* = 1/1). An improvement was also reported in oppositional behavior at one 2-month follow-up. Furthermore, scores on the Somatic and ADHD scales of the YSR dropped at the end of treatment, and the improvements were maintained over 2-, 6- and 12- months post-treatment. In studies that examined changes in irritability (Irritation scale on the Chronic Sleep Reduction Questionnaire), a significant reduction was evident in one study but not in another study. In this other study, a reduction in irritability, however, emerged from the post-treatment to 2-month follow-up. Finally, in a study that included children with ASD there was a drop in sleep anxiety on the Children's Sleep Habit Questionnaire (Owens et al., 2000) from baseline to post-treatment and follow-up.

The only study that examined changes in cognition from pre- to post-treatment found significantly increased efficiency of visual-spatial processing, as well as better selective attention and phonological working memory. No significant changes were detected in executive skills, language, episodic memory, and visual working memory. Improvements in visual processing, selective attention and working memory correlated with a reduction in WASO duration on sleep diaries. Improvements in selective attention, however, were related to an increase in WASO duration.

##### 3.4.2.2 Parents

Parental sleep, mood, and quality of life were assessed in 1 study each. Significant improvements were recorded in maternal SOL, WASO, TST, and subjective sleep ratings on completion of intervention, and were maintained (SOL, WASO, but not TST) on follow-up. Mood and quality of life improved during treatment relative to baseline and on completion of treatment/8-week follow-up, respectively. Fatigue reduced in two studies following treatment delivered remotely.

### 3.5 Quality ratings

**Figure 2:**
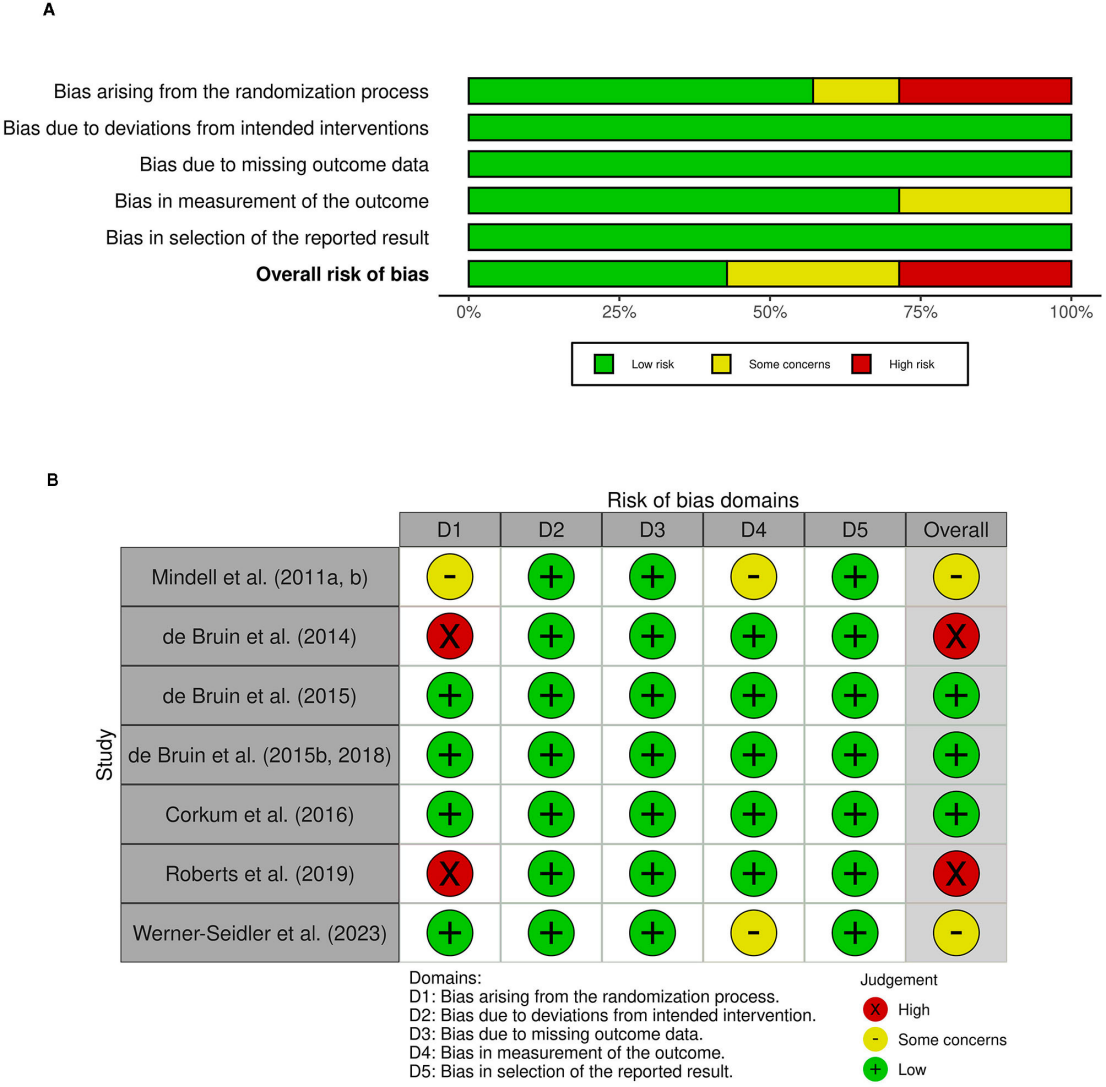
Risk of bias ratings **(A)** across studies: overall risk of bias and risk for each domain and **(B)** for each study on each domain.

Ratings on the risk of bias domains (randomization process, deviations from intended interventions, missing outcome data, measurement of the outcome, selection of the reported result) across studies and per individual study are summarized in Figures 2A, B, respectively.

#### 3.5.1 Randomization process

Across studies, we found risk of bias to be highest for the randomization process, having been assessed as high in two studies, having some concerns in one study, and evaluated as low in the remaining four studies. The key component of concern pertained to reporting of specific procedures used for the allocation of participations to an intervention group. While five studies reported using a random sequence that was specifically detailed, two studies provided no information regarding the random sequence generation beyond describing the study as an RCT. Further, randomization sequence was judged to be concealed until participants were enrolled into the study for four studies.

#### 3.5.2 Deviations from intended interventions (effect of assignment to intervention)

Across all studies, we found that risk of bias was low.

#### 3.5.3 Missing outcome data (effect of adhering to intervention)

Risk of bias in the domain of missing outcome data was judged to be low for all six studies. There was data available for all, or nearly all, participants, or significant missingness in data was accounted for by sensitivity analyses or other methods addressing for bias.

#### 3.5.4 Measurement of the outcome

Risk of bias in the measurement of the outcome was deemed to be low in five studies and having some concerns in two study. All studies used appropriate methods of measuring primary sleep outcome(s) across groups. As the outcome assessors were the participants or their parents, scores obtained on subjective outcome measures (i.e., questionnaires) could have been biased. However, subjective sleep measures were supplemented by an objective measure (actigraphy) in five of six studies. Actigraphy recordings were unlikely to have been substantially influenced by the intervention received. Thus, the overall risk was judged to be low for studies that used a combination of subjective and objective methods to assess sleep outcomes. Two study, however, relied solely on subjective measures, raising some concerns for risk of bias.

#### 3.5.5 Selection of the reported result

Only one of the seven studies included in the systematic review had a registered protocol outlining a pre-specified data analysis plan (Werner-Seidler et al., 2023). However, following contact with authors, all the remaining studies were confirmed to have pre-specified data analysis plan. As such, all studies were judged to be low risk within this domain.

## 4 Discussion

Insomnia and behavioral sleep disturbances are common in the general child and adolescent population (Chung et al., 2014; Medalie et al., 2021) and can be effectively treated with behavioral therapies (Jacobs et al., 2004; Meltzer and Mindell, 2014). The evidence base for effectiveness of pediatric behavioral insomnia treatments largely stems from face-to-face treatments delivered by trained therapists (Meltzer and Mindell, 2014). Such treatments are associated with high costs, limited access, and insufficient capacity relative to high needs for treatment. Thus, it is paramount to develop an evidence base for remotely delivered behaviourally based treatments for pediatric insomnia, which are scalable and can be accessed readily. Our systematic review included 7 RCTs (9 publications) that delivered behavioral therapy for insomnia/insomnia symptoms to pediatric populations remotely. Overall, our study provided preliminary support for the remotely delivered behavioral sleep interventions improving sleep pre- to post-treatment. The risk of bias ranged from low to high, with the highest risk of bias found for the randomization process. The review revealed the following main findings: all studies found significant improvements in sleep following remotely delivered treatments, across multiple, but not all, sleep outcomes. Studies that compared effectiveness of remotely delivered treatments to treatments delivered face-to-face provided mixed findings, albeit raised a possibility that remotely delivered treatments may be slightly less potent than treatments delivered face-to-face. Secondary gains were documented in children's mood from pre- to post- treatment. Secondary gains in cognition and parental wellbeing were understudied but found. No study reported on either academic skills or quality of life in children.

Our systematic review revealed that the evidence base for effectiveness of remotely delivered behavioral treatments for child and adolescent insomnia/insomnia symptoms is in its infancy, which is in sharp contrast with adult literature that has generated a robust body of evidence supporting effectiveness of remotely delivered CBT-I (Rios et al., 2019; for a review see Forma et al., 2022). Just like in adult studies, however, we found significant improvements in sleep following treatments delivered remotely, which were largely maintained over follow-ups, up to 12 months post-treatment. Nevertheless, significant improvements were not found on all sleep outcomes consistently. Across studies, total sleep quality and sleep efficacy improved, as measured by questionnaires, and sleep diaries and actigraphy, respectively. Improvements were also frequently found in sleep onset latency as well as in total sleep time and waking after sleep onset; though, on the last two sleep parameters improvements were less consistently found. No study examined changes in sleep regularity, which is important for sleep health across ages (Meltzer et al., 2021; Sletten et al., 2023). Selective improvements in insomnia symptoms raise a possibility that behavioral sleep interventions may not be equally effective for treatment of different symptoms of insomnia in children and adolescents. Nevertheless, the lack of improvement may also be due to differences in the severity of insomnia symptoms and to differences in treating populations (i.e., neurotypical vs. neurodiverse). In contrast to the prevailing evidence of improved sleep following treatments, two studies that involved neurodiverse participants reported opposite findings with respect to changes in one sleep parameter (ADHD, Corkum et al., 2016; ASD, Roberts et al., 2019). Sleep onset latency was significantly shorter on the questionnaire (improved) but significantly longer (worsened) and above the cut-off for clinically significant difficulties on actigraphy from pre- to post-treatment but improved on follow-up in participants with ADHD (Corkum et al., 2016). The authors attributed these unusual findings to the low actigraphy adherence in the treatment group (45%, *n* = 14) relative to the waitlisted group (70%, *n* = 21). In a study by Roberts et al. (2019), waking improved on the questionnaire but deteriorated on actigraphy in participants with ASD in the online group. In the face to face group, waking improvement on both, the questionnaire and actigraphy. The authors have not offered explanations as to why the increase in the wake after sleep onset increased in the online group but not in the face-to-face-group. With respect to a difference in findings between questionnaire and actigraphy, the authors stated that changes in wakings may not have been perceived by parents. The increase of time needed to fall asleep and in wakening found in these two studies, however, also raises concerns about a possible worsening of symptoms following remotely delivered behavioral treatments for pediatric insomnia and highlights the need to examine outcome at an individual as well as group level.

It is encouraging to see that remotely delivered behavioral treatments may be effective in improving insomnia symptoms in children. Nevertheless, it is important to determine whether effectiveness of treatments delivered remotely is comparable to treatments delivered face-to-face. Only three RCTs compared effectiveness of treatments delivered remotely with treatments delivered face-to-face, and only one of these studies compared two modes of treatment delivery with a waitlisted condition (de Bruin et al., 2015b). Remotely delivered treatment was found to be slightly less effective (sleep efficacy, sleep onset latency and waking after sleep onset measured by actigraphy and/or sleep diaries) in two studies (de Bruin et al., 2014, 2015b) involving neurotypical participants or largely comparable to treatments delivered face-to-face in a study involving participants with ASD (Roberts et al., 2019). In contrast to our systematic review, a recent network meta-analysis found that digitally delivered CBT-I was more effective relative to CBT-I delivered face-to-face (Forma et al., 2022). On detailed examination of the study by Forma et al. (2022), we noticed that increased effectiveness of the digitally delivered CBT-I was found for scores obtained on The Insomnia Severity Index (ISI; Bastien et al., 2001). In contrast, patients reported no significant changes in either sleep onset latency or wake after sleep onset. Hence, the difference in findings could possibly, in part, be explained by a difference in measures used; no study included in our review used the ISI, which is not surprising, as the ISI was only recently adapted for use with children: pediatric insomnia severity index (PISI; Byars et al., 2017). It is also possible, however, that the difference in finding may be related to the differences in populations included in the reviews; while our review included child and adolescent studies, a review by Forma et al. (2022) included adult studies.

Possible secondary gains were not routinely assessed in the RCTs that were included in our study. Changes in mood were most commonly evaluated. All studies that assessed mood found significant improvements in certain aspects of child's mood either on completion of treatment or on follow-up. The reported improvements in mood ranged from generalized improvement in internalizing behavioral symptoms that are reflective of mood disturbances (i.e., anxiety, depression, social withdrawal; de Bruin et al., 2018) to specific improvement in sleep anxiety (Roberts et al., 2019), as rated by parents. Our findings are consistent with adult literature, which showed that digital CBTi resulted in improvements in both sleep and mood (Cheng et al., 2019; Lee et al., 2023; anxiety and depression symptoms). While it was encouraging to find significant improvements in ADHD symptoms and select aspects of cognition in children as well as in sleep and parent wellbeing, these outcomes were examined in one study only each.

Our review has several limitations. Studies included in our review varied with respect to the treatments used, age of participants, type of participants (neurotypical or neurodiverse) and instruments employed to validate outcomes. However, one of the treatment programs, Sleep Smart, was evaluated in 3 out of 7 RCTs. Nevertheless, all 3 studies were conducted by the same research group. While findings of studies that used Sleep Smart are encouraging, further independent validations are required. Participants who received treatments in the studies that were included in our review ranged in their developmental stages from infants, school aged children to adolescents. Such a wide age-range of participants makes it difficult to synthesize the findings, as types of sleep difficulties and treatment delivery (i.e., parental involvement in treatment) change from infancy to adolescence. Furthermore, studies also differed with respect to types of participants, with two studies including neurodiverse participants (ADHD, Corkum et al., 2016; ASD, Roberts et al., 2019) whose developmental features and needs may have impacted treatment outcomes. In addition, heterogeneity was also noticed in subjective measures of sleep, which was necessitated (at least in part) by participants' age. In contrast to subjective measures of sleep, actigraphy was consistently used to measure sleep objectively. Surprisingly, given the multiple between-study differences, improvements in sleep were found across studies. In part, these encouraging findings may be due to treatment programs being based on trialed and tested principles or programs previously delivered face-to-face. For example, all programs included psychoeducation about sleep and specific behavioral and cognitive strategies to improve sleep. Moreover, we noticed that the content and techniques used in interventions were carefully matched to developmental, and special needs of the participants. In addition to heterogeneity, it is important to notice that several studies included in our review had a small number of participants (*n* < 50). Furthermore, our review was also limited to studies published in the English language, included only a small number of studies, and did not perform meta-analysis due to the small sample of heterogeneous studies. What is more, all studies included in this review were conducted in developed, western cultures. This is important, as acceptance and outcomes of remotely delivered behavioral interventions for sleep difficulties may not be the same in other cultures, as sleep conditions, sleep practices and behaviors differ between cultures (Airhihenbuwa et al., 2016; George et al., 2021).

Overall, our systematic review provides a preliminary evidence base for effectiveness of remotely delivered behavioral treatments for insomnia symptoms and behavioral sleep disturbances in children and adolescents. Our findings are encouraging, albeit should be interpreted with caution as they are based on a small number of heterogeneous studies, with treatment delivery ranging from fully automated to those that were delivered by sleep therapists remotely and supplemented by materials available online. For scalability, fully automated treatments are the most suitable. Nevertheless, further research is needed to establish whether effectiveness of remotely delivered behavioral insomnia treatments is comparable to face-to-face treatments in the general as well as in clinical populations of children and adolescents, and whether these treatments improve not only sleep but also daily functioning and wellbeing of children and their caregivers. Moreover, it may be important to further examine which insomnia symptoms could be effectively treated with remotely delivered behavioral therapies and personalize treatments to match the types of insomnia symptoms and developmental levels of participants.

## Author contributions

SL: Conceptualization, Formal analysis, Funding acquisition, Investigation, Methodology, Project administration, Resources, Supervision, Validation, Writing – original draft. TC: Data curation, Formal analysis, Investigation, Validation, Visualization, Writing – original draft.
