# Supplementary material for: Outcomes of remotely delivered behavioral insomnia interventions for children and adolescents: systematic review of randomized controlled trials
Source: Front Sleep. 2024 Jan 11;2:1261142. doi: 10.3389/frsle.2023.1261142 (PMC12713916; doi:10.3389/frsle.2023.1261142)
Supplement: Supplementary file 1 [file Data_Sheet_1.docx]

Supplementary Material

| **MEDLINE searched via Ovid on 18 October 2022 and updated on 1 November 2023 (from 1946 to November 1, 2023)**   1. exp Sleep/ 2. exp Sleep Wake Disorders/ 3. exp “Sleep Initiation and Maintenance Disorders”/ 4. sleep disturbance.mp 5. insomnia.mp 6. 1 or 2 or 3 or 4 or 5 7. intervention.mp 8. program.mp 9. therapy.mp 10. treatment.mp 11. non-pharmacological.mp 12. behavioural.mp 13. management.mp 14. trial.mp 15. randomised control trial.mp 16. CBT.mp 17. cognitive.mp 18. 7 or 8 or 9 or 10 or 11 or 12 or 13 or 14 or 15 or 16 or 17 19. electronic.mp 20. app.mp 21. computer.mp 22. cyber.mp 23. cyberspace.mp 24. internet.mp 25. net.mp 26. online.mp 27. virtual.mp 28. web.mp 29. e-health.mp 30. mobile 31. smartphone 32. tele-health 33. mobile applications.mp 34. 19 or 20 or 21 or 22 or 23 or 24 or 25 or 26 or 27 or 28 or 29 or 30 or 31 or 32 or 33 35. 6 and 18 and 34 36. limit 35 to (english language and humans and "all child (0 to 18 years)") |
| --- |
| **PsychINFO searched via Ovid on 18 October 2022 and updated on 28 October 2023 (from 1806 to October 28, 2023)**   1. exp Sleep/ 2. exp Sleep Wake Disorders/ 3. sleep disturbance.mp 4. insomnia.mp 5. 1 or 2 or 3 or 4 6. intervention.mp 7. program.mp 8. therapy.mp 9. treatment.mp 10. non-pharmacological.mp 11. behavioural.mp 12. management.mp 13. trial.mp 14. randomised control trial.mp 15. CBT.mp 16. cognitive.mp 17. 6 or 7 or 8 or 9 or 10 or 11 or 12 or 13 or 14 or 15 or 16 18. electronic.mp 19. app.mp 20. computer.mp 21. cyber.mp 22. cyberspace.mp 23. internet.mp 24. net.mp 25. online.mp 26. virtual.mp 27. web.mp 28. e-health.mp 29. mobile 30. smartphone 31. tele-health 32. mobile applications.mp 33. 18 or 19 or 20 or 21 or 22 or 23 or 24 or 25 or 26 or 27 or 28 or 29 or 30 or 31 or 32 34. 5 and 17 and 3 35. limit 34 to (peer reviewed journal and (childhood <birth to 12 years> or adolescence <13 to 17 years>)) |
| **Cochrane Central Register of Controlled Trials (CENTRAL) searched on 17 October 2022 and updated on 28 October 2023 (from 1998 to October 28, 2023)**   1. exp Sleep/ 2. exp Sleep Wake Disorders/ 3. sleep disturbance.mp 4. insomnia.mp 5. 1 or 2 or 3 or 4 6. intervention.mp 7. program.mp 8. therapy.mp 9. treatment.mp 10. non-pharmacological.mp 11. behavioural.mp 12. management.mp 13. trial.mp 14. randomised control trial.mp 15. CBT.mp 16. cognitive.mp 17. 6 or 7 or 8 or 9 or 10 or 11 or 12 or 13 or 14 or 15 or 16 18. electronic.mp 19. app.mp 20. computer.mp 21. cyber.mp 22. cyberspace.mp 23. internet.mp 24. net.mp 25. online.mp 26. virtual.mp 27. web.mp 28. e-health.mp 29. mobile 30. smartphone 31. tele-health 32. mobile applications.mp 33. 18 or 19 or 20 or 21 or 22 or 23 or 24 or 25 or 26 or 27 or 28 or 29 or 30 or 31 or 32 34. exp Child/ 35. exp Adolescent/ 36. 34 or 35 37. 5 and 17 and 33 and 36 |

**Supplementary Table 1.** Search strategy

**Identification of studies via databases and registers**

Records identified from:

Databases (n = 1154)

PsychINFO (n = 266)

Medline (n = 780)

Cochrane CENTRAL (n = 108)

In-text references (n = 6)

Records removed *before screening*:

Duplicate records removed

(n = 252)

**Identification**

Records screened

(n = 908)

Records excluded

(n = 834)

Reports excluded:

Mean age > 18 years (n = 19)

Not sample with insomnia or insomnia symptoms (n = 10)

Not RCT (n = 18)

Not intervention study (n = 5)

Not remote intervention (n = 6)

Not behavioural sleep intervention (n = 7)

Paper not yet published (n = 1)

**Screening**

Reports assessed for eligibility

(n = 74)

Studies included in systematic review (n = 8)

**Included**

**Supplementary Figure 1.** PRISMA Flowchart of studies selection process
